# Platelet-to-lymphocyte ratio as a predictive factor of complete pathologic response to neoadjuvant chemotherapy in breast cancer

**DOI:** 10.1371/journal.pone.0207224

**Published:** 2018-11-14

**Authors:** Javier Cuello-López, Ana Fidalgo-Zapata, Laura López-Agudelo, Elsa Vásquez-Trespalacios

**Affiliations:** 1 Clinical Oncology Group, Fundación Colombiana de Cancerología-Clínica Vida, Medellín, Colombia; 2 Breast Surgeon Fellowship Program, School of Medicine, CES University, Medellín, Colombia; 3 Fundación Colombiana de Cancerología-Clínica Vida, Medellín, Colombia; 4 Department of Clinical Epidemiology, School of Medicine, CES University, Medellín, Colombia; University of Kentucky, UNITED STATES

## Abstract

Response to neoadjuvant chemotherapy in breast cancer patients is of prognostic value in determining short- and mid-term outcomes. Inflammatory biomarkers, such as platelet-to-lymphocyte ratio (PLR) and neutrophil to lymphocyte ratio (NLR), have been proposed as predictive factors of response to neoadjuvant chemotherapy. Currently, there are no studies in Colombian patients reporting the role of inflammatory biomarkers as response predictors in patients receiving neoadjuvant chemotherapy. Therefore, in this study we performed a cross-sectional study and analyzed the association between inflammatory biomarkers and pCR (pathological complete response) in patients diagnosed with breast cancer–of different molecular subtypes- and treated with neoadjuvant chemotherapy. A total of 288 patients were included in the study, with a median age of 51 years old. Disease was locally advanced in 83% of the participants, and 77.7% had compromised lymph nodes. In our cohort, the most frequent tumor molecular subtype was luminal B/Her2- (27.8%) followed by triple negative [TN] (21.5%), luminal B/Her2+ (19.8%), Her2-enriched (16%) and luminal A (13.5%). PLR was not associated with age, menopausal status, baseline tumor size, histologic grade, axillary lymph node involvement, disease stage, estrogen receptor status, or Ki67; however, complete pathological response was significantly higher in the low PLR group (PLR<150) compared with the high PLR group (35.1% Vs. 22.2%, p = 0.03). In addition, Her2-enriched tumors achieved the highest pCR rates (65%), followed by TN (34%) tumors. Our results suggest that breast cancer patients with low platelet-to-lymphocyte ratio (PLR <150), treated with neoadjuvant chemotherapy achieve higher complete pathological response, independently of primary tumor molecular subtype.

## Introduction

Pathological complete response (pCR) to neoadjuvant chemotherapy in breast cancer patients is of prognostic value in determining short-, and mid-term outcomes, and is one of the main objectives in current ongoing studies assessing the potential benefit of neoadjuvant chemotherapy [1–3].

In the United States, the Food and Drug Administration (FDA) has recommended inclusion of pathologic complete response (pCR) as a main requirement for the accelerated approval of drugs used for neoadjuvant therapy in early-stage, high-risk breast cancer [4, 5]. However, achieving a pCR after neoadjuvant therapy is associated with several pathologic and biologic factors such as histologic grade, hormone receptor status, and expression of Her2 and Ki67, among others. Among them, while luminal A tumors have been reported to be the least likely to achieve pCR after neoadjuvant therapy, followed by luminal B tumors–which show a moderate response-, Her2-enriched and TN tumors exhibit the highest pCR rates [6, 7].

It is well recognized that systemic inflammation plays an important role in promoting tumor progression. Many studies have shown that elevated inflammatory markers, such as neutrophil/lymphocyte ratio (NLR) and platelet/lymphocyte ratio (PLR), are associated with poor prognosis in patients with different solid malignancies. Also NLR and PLR may be related to chemosensitivity [8–14].

Currently, there are no studies from Colombia reporting the role of inflammatory biomarkers (NLR, PLR, etc) as response predictors in patients receiving neoadjuvant chemotherapy. Therefore, in this study we have analyzed the association between biomarkers (NLR, PLR) and pCR in patients diagnosed with breast cancer of different molecular subtypes and treated with neoadjuvant chemotherapy.

## Materials and methods

### Patients and study design

This study was approved by Institutional Ethics Committee of the Fundación Colombiana de Cancerologia-Clínica Vida. All data were fully anonymized before we accessed them. The Institutional Ethics Committee waived the requirement for informed consent.

This was a cross-sectional study in breast cancer patients treated with neoadjuvant therapy at our institution, *Fundación Colombiana de Cancerología-Clínica Vida*, in Medellín, Colombia between January 2013 and December 2016. Patient data were collected from electronic databases.

Patients receiving neoadjuvant therapy of anthracycline and/or taxanes sequential regimens were included in this study. Patients with Her2-expressing tumors were neoadjuvantly-treated with trastuzumab. Patients not included in the study were those with bilateral breast cancer, inflammatory breast carcinoma; those that had received <3 cycles of neoadjuvant therapy, documented acute infectious process, pregnancy, pre-operative diagnosis of chronic disease including chronic hepatic disease, terminal renal disease, or inflammatory diseases such as systemic lupus erythematous. Patients with inadequate disease staging were also excluded from the study.

### Variables

Patients (variables: age, cell counts, tumor characteristics) data were collected and analyzed from hemograms performed (less than 1 month) before initiating neoadjuvant chemotherapy. Platelet-to-lymphocyte ratio (PLR) was calculated as the ratio of the absolute platelet count to absolute lymphocyte count. According to previous studies, a value of PLR of 150 was established as a cut-off point to discriminate two groups: low PLR (<150) and high PLR (>150) [12, 15, 16].

Menopausal status was defined by any of the following criteria: age ≥ 60 years, bilateral oophorectomy, amenorrhea ≥ 1 year (in the absence of chemotherapy, tamoxifen, or ovarian suppression), or levels of FSH, LH or estradiol in the reported ranges for post-menopausal status.

Expression of estrogen receptor (ER) and progesterone receptor (PR) was assessed by immunohistochemistry. Depending on the case, Her2 expression was analyzed by immunohistochemistry or fluorescent in situ hybridization (FISH). Her2 was considered positive by immunohistochemistry for all 3+ cases, or if expression of Her2 was at least two-fold compared to expression of CEP17 in tumor cells [17].

Intrinsic subtypes were classified as follows: luminal A (ER positive, PR positive, Her2 negative, and Ki67<20%); luminal B- (ER positive, PR positive or negative, Her2 negative, Ki67 ≥20%); luminal B+ (ER positive, PR positive or negative, Her2 positive, independently of Ki67 value); Her2-enriched (ER negative, PR negative, Her2 positive, independently of Ki67 value); and triple negative (ER negative, PR negative, Her2 negative, independently of Ki67 value) [18].

Pathological complete response was defined as the absence of invasive disease in the breast and axilla (ypT0ypN0 or ypT0/is ypN0).

### Statistical analysis

The main purpose of this study was to assess the role of PLR before neoadjuvant therapy as a potential predictive marker of pathological response in breast cancer patients with operable disease. The association between PLR and the clinicopathologic variables was assessed by Chi-square and Fisher tests.

Data were analyzed using SPSS software (Windows version 21). Quantitative variables are shown as mean or median with their corresponding dispersion measures according to the variables distribution. Qualitative variables were represented as percentages. Comparison of averages was done by Student’s t-test for independent samples, or by the Mann-Whitney U-test, as required. Groups were compared by means of Chi-square and Fisher test for categorical variables, while ANOVA and Kruskal-Wallis were used for comparison among groups of continuous variables (depending on the distribution of the continuous variables). Statistical significance is achieved if p <0.05.

## Results

A total of 288 patients were included in this study. The median age was 51 years (range 27–85 years old), being 58% of the patients between 40–59 years of age. Disease was locally advanced in 239 (83%) patients (21,2% IIB, 25% IIIA, 32% IIIB, and 4,8% IIIC), and most patients, 224 of 288 (77.7%), presented compromised lymph nodes (N1-N3). Tumor subtypes in our population were distributed as follows: 13.5% luminal A tumors, 27.8% luminal B/Her2 negative, 19.8% luminal B/Her2 positive, 21.5% TN, and 16% Her2-enriched (Table 1).

**Table 1:** Baseline characteristics.

**Characteristics**	N = 288
**Age at diagnosis, median in years (range)**	51 (27–85)
**Range, ages (years)**	**n (%)**
<35 years	22 (6.2)
35–39	27 (11.1)
40–49	78 (27.1)
50–59	91 (31.6)
60–69	50 (17.4)
≥70	20 (6.6)
**Menopausal status**	**n (%)**
Premenopausal	174 (60.4)
Postmenopausal	114 (39.6)
**Tumor size**	**n (%)**
TX	3 (1.0)
T1	7 (2.4)
T2	107 (37.1)
T3	71 (24.6)
T4	100 (34.7)
**Lymph nodes**	**n (%)**
N0	64 (22.2)
N1	124 (43)
N2	86 (29.8)
N3	14 (4.8)
**TNM**	**n (%)**
IIA	49 (17)
IIB	61 (21.2)
IIIA	72 (25)
IIIB	92 (32)
IIIC	14 (4.8)
**Histologic subtype**	**n (%)**
Ductal	274 (95.1)
Lobular	6 (2.1)
Other	8 (2.8)
**Tumor grade**	**n (%)**
G1	21 (7.3)
G2	131 (45.5)
G3	116 (40.3)
Unknown	20 (6.9)
**Estrogen receptor status**	**n (%)**
Positive	178 (61.8)
Negative	110 (38.2)
**Progesterone receptor status**	**n (%)**
Positive	142 (49.3)
Negative	146 (50.7)
**HER 2**	**n (%)**
Positive	103 (35.7)
Negative	184 (64)
Unknown	1 (0.3)
**Biological subtype (IHC4)**	**n (%)**
Luminal A	39 (13.5)
Luminal B, HER 2-	80 (27.8)
Luminal B, HER 2+	57 (19.8)
Her 2 enriched	46 (16)
Triple Negative	62 (21.5)
**Neoadjuvant Chemotherapy regimen**	**n (%)**
Anthracycline-based only	11 (3.8)
Taxanes-based only	29 (10.1)
Anthracycline and taxane based	248 (86.1)
**Pathological response**	**n (%)**
pCR	89 (30.9)
No pCR	199 (69.1)
**Characteristics**	N = 288
**Age at diagnosis, median in years (range)**	51 (27–85)
**Range, ages (years)**	**n (%)**
<35 years	22 (6.2)
35–39	27 (11.1)
40–49	78 (27.1)
50–59	91 (31.6)
60–69	50 (17.4)
≥70	20 (6.6)
**Menopausal status**	**n (%)**
Premenopausal	174 (60.4)
Postmenopausal	114 (39.6)
**Tumor size**	**n (%)**
TX	3 (1.0)
T1	7 (2.4)
T2	107 (37.1)
T3	71 (24.6)
T4	100 (34.7)
**Lymph nodes**	**n (%)**
N0	64 (22.2)
N1	124 (43)
N2	86 (29.8)
N3	14 (4.8)
**TNM**	**n (%)**
IIA	49 (17)
IIB	61 (21.2)
IIIA	72 (25)
IIIB	92 (32)
IIIC	14 (4.8)
**Histologic subtype**	**n (%)**
Ductal	274 (95.1)
Lobular	6 (2.1)
Other	8 (2.8)
**Tumor grade**	**n (%)**
G1	21 (7.3)
G2	131 (45.5)
G3	116 (40.3)
Unknown	20 (6.9)
**Estrogen receptor status**	**n (%)**
Positive	178 (61.8)
Negative	110 (38.2)
**Progesterone receptor status**	**n (%)**
Positive	142 (49.3)
Negative	146 (50.7)
**HER 2**	**n (%)**
Positive	103 (35.7)
Negative	184 (64)
Unknown	1 (0.3)
**Biological subtype (IHC4)**	**n (%)**
Luminal A	39 (13.5)
Luminal B, HER 2-	80 (27.8)
Luminal B, HER 2+	57 (19.8)
Her 2 enriched	46 (16)
Triple Negative	62 (21.5)
**Neoadjuvant Chemotherapy regimen**	**n (%)**
Anthracycline-based only	11 (3.8)
Taxanes-based only	29 (10.1)
Anthracycline and taxane based	248 (86.1)
**Pathological response**	**n (%)**
pCR	89 (30.9)
No pCR	199 (69.1)

pCR: pathological complete response

In this study, 89 (30.9%) patients achieved pathological complete response. Tumor subtypes with highest response rates were Her2-enriched (65%) and TN (34%).

PLR was calculated for 272 patients and using a PLR value cut-off point of 150, the group was dichotomized into high (PLR>150) and low (PLR<150). PLR ranged between 50.84 and 1437.5 (mean: 146.05; median: 128.1; standard deviation: 109.38). The high PLR group included 90 patients (33%), and 182 patients (67%) formed the low PLR group (Table 2).

**Table 2:** Patient characteristics by PLR prior to neoadjuvant chemotherapy.

Characteristics	High PLR (≥150)	Low PLR (<150)	P-value
	n = 90 (33%)	n = 182 (67%)	
**Age, median in years (range)**	**50.3 (29–85)**	**51.5 (27–80)**	**0.415**
**Age range in years**			**0.611**
**<35**	**10%**	**5%**	
**35–39**	**8.9%**	**12.6%**	
**40–49**	**28.9%**	**27.4%**	
**50–59**	**27.8%**	**31.9%**	
**60–69**	**17.8%**	**16%**	
**= >70**	**6.6%**	**7.1%**	
**Menopausal status**			**0.209**
**Premenopausal**	**66.7%**	**58.8%**	
**Postmenopausal**	**33.3%**	**41.2%**	
**Histologic subtype**			**0.454**
**Ductal**	**96.7%**	**94%**	
**Lobular**	**2.22%**	**2.2%**	
**Other**	**1.11%**	**3.8%**	
**Histologic grade**			**0.229**
**Unknown**	**10%**	**5%**	
**Grade 1**	**7.8%**	**7.1%**	
**Grade 2**	**47.8%**	**42.9%**	
**Grade 3**	**34.4%**	**45%**	
**T stage (clinical)**			**0.622**
**Tx**	**1.1%**	**1.1%**	
**T1**	**2.2%**	**2.2%**	
**T2**	**33.4%**	**38.4%**	
**T3**	**22.2%**	**27%**	
**T4**	**41.1%**	**31.3%**	
**N stage (clinical)**			**0.668**
**N0**	**24.4%**	**20.3%**	
**N1**	**37.8%**	**45.6%**	
**N2**	**32.2%**	**29.1%**	
**N3**	**5.6%**	**5%**	
**Overall Staging**			**0.570**
**IIA**	**15.6%**	**16.4%**	
**IIB**	**22.2%**	**20.9%**	
**IIIA**	**20%**	**28.6%**	
**IIIB**	**36.7%**	**29.1%**	
**IIIC**	**5.5%**	**5%**	
**ER status**			**0.282**
**Positive**	**65.6%**	**58.8%**	
**Negative**	**34.4%**	**41.2%**	
**H-score ER (0–300)**	**139.8**	**129.7**	**0.565**
**PR status**			**0.008**
**Positive**	**55.6%**	**44.5%**	
**Negative**	**44.4%**	**55.5%**	
**H-score PR (0–300)**	**94**	**74**	**0.198**
**Her2 status**			**<0.001**
**Positive**	**21.1%**	**44%**	
**Negative**	**77.8%**	**56%**	
**Unknown**	**1.1%**	**0%**	
**%Ki67 (median)**	**38.5%**	**38.2%**	**0.941**
**Molecular subtype**			**0.009**
**Luminal A**	**17%**	**9.9%**	
**Luminal B/Her2 (-)**	**34.1%**	**25.8%**	
**Luminal B/Her2 (+)**	**13.6%**	**23.6%**	
**Her2 enriched**	**8%**	**20.3%**	
**Triple negative**	**26.1%**	**20.3%**	
**Neoadjuvant chemotherapy regimen**			
**Anthracycline-based only**	**3.3%**	**3.9%**	**0.607**
**Taxanes-based only**	**7.8%**	**11.5%**	
**Anthracycline and taxane based**	**88.9%**	**84.6%**	
**Pathological response**			**0.03**
**pCR**	**22.2%**	**35.1%**	
**No-pCR**	**77.8%**	**64.9%**	

High PLR values were significantly associated with PR expression (p = 0.008). Furthermore, in Her2 positive cases low PLR values were more frequently found than high PLR (p<0.001). In the subgroup analysis, Her2-enriched, which are related to higher pCR, were associated to a greater extent with low PLR. Because of this reason, we performed a multivariate analysis in order to rule out Her2 status as a confusion variable in relation to PLR and pCR. The results of this analysis support PLR as an independent predictive factor of pCR, independent of Her2 status (Table 3). PLR levels were not associated with age, menopausal status, baseline tumor size, histologic grade, axillary lymph node involvement, disease stage, estrogen receptor status, or Ki67. Pathological complete response was significantly higher in the low PLR group compared with the high PLR group (35.1% Vs. 22.2%, p = 0.03)

**Table 3:** Association between PLR and pCR by Her2 status.

Characteristics		pCR	No-pCR	P-value
**Her2 positive**	High PLR (≥150)	10 (10.1%)	9 (9.1%)	0.992
	Low PLR (<150)	42 (42.4%)	38 (38.4%)	
**Her2 negative**	High PLR (≥150)	10 (5.8%)	60 (34.9%)	0.228
	Low PLR (<150)	22 (12.8%)	80 (46.5%)	

## Discussion

Complete pathological response is a strong prognostic factor in breast cancer patients[7]. Recent studies in the neoadjuvant setting have included pCR as a primary objective to predict long-term clinical outcomes such as disease-free survival (DFS) and overall survival (OS) [1, 19]. This has been observed mainly for the aggressive Her2 positive (non-luminal) and TN breast cancers [1, 3].

In this study, pCR was 30.9%; being comparable to previously reported studies based on anthracycline and taxane-based regimens, which reported pCR rates ranging from 20 to 40% [3]. Furthermore, pCR is associated with morphologic and biologic tumor attributes, such as hormone receptor status, and Her2 receptor status, among others.

Recently, Xiaoxian et al, reported that patients with Her2-enriched and TN tumors achieve pCR rates of 58.2% and 47.4% respectively, considerably higher than those achieved by luminal tumors (27.8%) [20]. In agreement with those results, our study also shows that Her2-enriched tumors achieved the highest pCR rates (65%), followed by TN (34%) tumors.

### PLR ratio and clinicopathologic factors

Previous studies have described a role for platelets in cancer growth and progression, by releasing VEGF-A and thus activating angiogenesis, and by their interaction with inflammatory mediators such as interleukins and myeloid metalloproteins [21, 22]. This has prompted multiple studies assessing the prognostic value of platelet counts and ratio to other immune cells -including lymphocytes and neutrophils- in the context of breast cancer [11, 13, 14].

The study by Asano et al. in a Japanese population, found an association between a low PLR ratio (cut-off: PLR <150), age (> 56 years) and postmenopausal status (p = <0.001) [15]. Krenn-Pilko, et al, showed that high PLR (cut-off: PLR > 292) correlated with lymph node involvement and high tumor grade (p = <0.05), while it was not found to be associated with other clinicopathologic variables [10]. On another hand, the study by Koh et al. reported an association of high PLR (cut-off: PLR > 215) with age (> 50 years, p = <0.01) and tumor size (>5cm, p = <0.01), while no association was found with lymph node involvement or tumor grade (p = 0.091 and p = 0.06, respectively) [9].

In this study, PLR of Colombian patients receiving neoadjuvant therapy was not associated with clinicopathologic features including age (p = 0.611), menopausal status (p = 0.209), tumor grade (p = 0.229), tumor size (p = 0.622), lymph node involvement (p = 0.680), and stage (p = 0.570). Altogether, studies by others and us suggest that disease heterogeneity and population differences may have an effect on the association of PLR with clinicopathologic variables.

### PLR and pathological complete response

In 2011, Hanahan and Weinberg [23] proposed tumor-promoting inflammation as an enabling characteristic for cancer growth. Furthermore, by analyzing hemogram data (neutrophil lymphocyte, monocyte, and platelet count), multiple studies have reported the role of the immune response as a prognostic factor of solid tumor progression [24–27].

Tumor cells have been shown to induce synthesis of platelet stimulating factors that favor growth, invasion and metastasis of primary tumors by several mechanisms. Thus, peripheral blood platelet counts could be an indirect indicator of tumor activity [10, 28]. On the other hand, detection of high numbers of peripheral blood lymphocytes with antitumor activity–particularly CD8+ T cells- would be an indicator of tumor suppression activity. Thus, these data suggest that patients with low PLR would have high antitumor activity, better prognosis, and better chemotherapy response.

In the study by Asano and colleagues, PLR was reported as a predictive and prognostic biomarker in a cohort of triple negative breast cancer patients in the neoadjuvant setting, since patients with low PLR exhibited higher rates of pCR, DFS, and OS [16]. Rafee, et al. reproduced these observations a smaller cohort of breast cancer patients in which high PLR (≥138.19) was independently associated with poor response to neoadjuvant chemotherapy [12]. In agreement with those studies, our results showed that PLR was associated with pCR, being patients with low PLR (<150) the ones achieving higher rates of pCR (35.1% Vs. 22.2%, p = 0.03). Altogether, these data support the role of PLR as a predictive variable of pCR that is independent of tumor molecular subtype.

It has been proposed that tumors with greater neoantigen synthesis–such as TN and Her2-positive- may achieve a higher immune anti-tumor response by increasing numbers of peripheral lymphocytes, and thus potentiating the effect of neoadjuvant chemotherapy. This is supported by studies reporting tumor-infiltrating lymphocytes (TILs) as predictive biomarker of cPR in the neoadjuvant setting, especially in early-stage Her2-positive and TN tumors. In addition, TILs are of prognostic value, since high TILs number associates with improved overall survival, strongly supports the notion that activation of the immune system is of critical importance for survival outcome [20, 29–39].

In summary, Breast cancer patients with low platelet-to-lymphocyte ratio (PLR <150) treated with neoadjuvant chemotherapy achieved a higher complete pathological response, independently of tumor molecular subtype.

## Supporting information

S1 Table Results of crosstabs between overall variables and PLR index. (PDF)
