# Supplementary material for: Platelet-to-lymphocyte ratio as a predictive factor of complete pathologic response to neoadjuvant chemotherapy in breast cancer
Source: PLoS One. 2018 Nov 14;13(11):e0207224. doi: 10.1371/journal.pone.0207224 (PMC6235359; doi:10.1371/journal.pone.0207224)
Supplement: S1 Table — (PDF) [file pone.0207224.s001.pdf]

```
CROSSTABS
  /TABLES=RECNLR BY PR
  /FORMAT=AVALUE TABLES
  /STATISTICS=CHISQ RISK
  /CELLS=COUNT COLUMN
  /COUNT ROUND CELL.
```

Crosstabs

Notes

Output Created		25-oct-2016 14:24:38
Comments		
Input	Data	C:\Users\evasquez\Documents\2016\Mastologia\NRI.sav
	Active Dataset	DataSet1
	Filter	<none>
	Weight	<none>
	Split File	<none>
	N of Rows in Working Data File	288
Missing Value Handling	Definition of Missing	User-defined missing values are treated as missing.
	Cases Used	Statistics for each table are based on all the cases with valid data in the specified range(s) for all variables in each table.
Syntax		CROSSTABS<br>/TABLES=RECNLR BY PR<br>/FORMAT=AVALUE TABLES<br>/STATISTICS=CHISQ RISK<br>/CELLS=COUNT COLUMN<br>/COUNT ROUND CELL.
Resources	Processor Time	00:00:00,000
	Elapsed Time	00:00:00,000
	Dimensions Requested	2

Notes

Output Created		25-oct-2016 14:24:38
Comments		
Input	Data	C:\Users\evasquez\Documents\2016\Mastologia\NRI.sav
	Active Dataset	DataSet1
	Filter	<none>
	Weight	<none>
	Split File	<none>
	N of Rows in Working Data File	288
Missing Value Handling	Definition of Missing	User-defined missing values are treated as missing.
	Cases Used	Statistics for each table are based on all the cases with valid data in the specified range(s) for all variables in each table.
Syntax		CROSSTABS<br>/TABLES=RECNLR BY PR<br>/FORMAT=AVALUE TABLES<br>/STATISTICS=CHISQ RISK<br>/CELLS=COUNT COLUMN<br>/COUNT ROUND CELL.
Resources	Processor Time	00:00:00,000
	Elapsed Time	00:00:00,000
	Dimensions Requested	2
	Cells Available	174762

```
[DataSet1] C:\Users\evasquez\Documents\2016\Mastologia\NRI.sav
```

Case Processing Summary

	Cases					
	Valid		Missing		Total	
	N	Percent	N	Percent	N	Percent
RECNLR * PR	288	100,0%	0	,0%	288	100,0%

RECNLR * PR Crosstabulation

			PR		Total
			0	1	
RECNLR	1	Count	178	81	259
		% within PR	89,4%	91,0%	89,9%
	2	Count	21	8	29
		% within PR	10,6%	9,0%	10,1%
Total		Count	199	89	288
		% within PR	100,0%	100,0%	100,0%

Chi-Square Tests

	Value	df	Asymp. Sig. (2-sided)	Exact Sig. (2-sided)	Exact Sig. (1-sided)
Pearson Chi-Square	,166[a]	1	,684		
Continuity Correction[b]	,038	1	,845		
Likelihood Ratio	,169	1	,681		
Fisher's Exact Test				,833	,431
N of Valid Cases	288				

a. 0 cells (,0%) have expected count less than 5. The minimum expected count is 8,96.

b. Computed only for a 2x2 table

Risk Estimate

	Value	95% Confidence Interval	
		Lower	Upper
Odds Ratio for RECNLR (1 / 2)	,837	,356	1,970
For cohort PR = 0	,949	,747	1,206
For cohort PR = 1	1,134	,612	2,100
N of Valid Cases	288		

```
SORT CASES  BY Quart_age.
SPLIT FILE SEPARATE BY Quart_age.
```

```
CROSSTABS
  /TABLES=RECNLR BY PR
  /FORMAT=AVALUE TABLES
  /STATISTICS=CHISQ RISK
  /CELLS=COUNT COLUMN
  /COUNT ROUND CELL.
```

Crosstabs

Notes

Output Created		25-oct-2016 14:27:11
Comments		
Input	Data	C:\Users\evasquez\Documents\2016\Mastologia\NRI.sav
	Active Dataset	DataSet1
	Filter	<none>
	Weight	<none>
	Split File	Quart_age
	N of Rows in Working Data File	288
Missing Value Handling	Definition of Missing	User-defined missing values are treated as missing.
	Cases Used	Statistics for each table are based on all the cases with valid data in the specified range(s) for all variables in each table.
Syntax		CROSSTABS /TABLES=RECNLR BY PR /FORMAT=AVALUE TABLES /STATISTICS=CHISQ RISK /CELLS=COUNT COLUMN /COUNT ROUND CELL.
Resources	Processor Time	00:00:00,015
	Elapsed Time	00:00:00,015
	Dimensions Requested	2
	Cells Available	174762

[DataSet1] C:\Users\evasquez\Documents\2016\Mastologia\NRI.sav

Quart_age = 1

Case Processing Summary[a]

	Cases					
	Valid		Missing		Total	
	N	Percent	N	Percent	N	Percent
RECNLR * PR	18	100,0%	0	,0%	18	100,0%

a. Quart_age = 1

RECNLR * PR Crosstabulation[a]

			PR		Total
			0	1	
RECNLR	1	Count	13	3	16
		% within PR	92,9%	75,0%	88,9%
	2	Count	1	1	2
		% within PR	7,1%	25,0%	11,1%
Total		Count	14	4	18
		% within PR	100,0%	100,0%	100,0%

a. Quart_age = 1

Chi-Square Tests[c]

	Value	df	Asymp. Sig. (2-sided)	Exact Sig. (2-sided)	Exact Sig. (1-sided)
Pearson Chi-Square	1,004[a]	1	,316		
Continuity Correction[b]	,010	1	,920		
Likelihood Ratio	,854	1	,355		
Fisher's Exact Test				,405	,405

N of Valid Cases	18				

a. 3 cells (75,0%) have expected count less than 5. The minimum expected count is ,44.

b. Computed only for a 2x2 table

c. Quart_age = 1

Risk Estimate[a]

	Value	95% Confidence Interval	
		Lower	Upper
Odds Ratio for RECNLR (1 / 2)	4,333	,207	90,847
For cohort PR = 0	1,625	,398	6,628
For cohort PR = 1	,375	,067	2,096
N of Valid Cases	18		

a. Quart_age = 1

Quart_age = 2

Case Processing Summary[a]

	Cases					
	Valid		Missing		Total	
	N	Percent	N	Percent	N	Percent
RECNLR * PR	32	100,0%	0	,0%	32	100,0%

a. Quart_age = 2

RECNLR * PR Crosstabulation[a]

			PR		Total
			0	1	
RECNLR	1	Count	20	10	30
		% within PR	90,9%	100,0%	93,8%
	2	Count	2	0	2
		% within PR	9,1%	,0%	6,3%

Total	Count	22	10	32
	% within PR	100,0%	100,0%	100,0%

a. Quart_age = 2

Chi-Square Tests[c]

	Value	df	Asymp. Sig. (2-sided)	Exact Sig. (2-sided)	Exact Sig. (1-sided)
Pearson Chi-Square	,970[a]	1	,325		
Continuity Correction[b]	,039	1	,844		
Likelihood Ratio	1,559	1	,212		
Fisher's Exact Test				1,000	,466
N of Valid Cases	32				

a. 2 cells (50,0%) have expected count less than 5. The minimum expected count is ,63.

b. Computed only for a 2x2 table

c. Quart_age = 2

Risk Estimate[a]

	Value	95% Confidence Interval	
		Lower	Upper
For cohort PR = 0	,667	,518	,859
N of Valid Cases	32		

a. Quart_age = 2

Quart_age = 3

Case Processing Summary[a]

	Cases					
	Valid		Missing		Total	
	N	Percent	N	Percent	N	Percent
RECNLR * PR	78	100,0%	0	,0%	78	100,0%

a. Quart_age = 3

RECNLR * PR Crosstabulation[a]

			PR		
			0	1	Total
RECNLR	1	Count	49	22	71
		% within PR	90,7%	91,7%	91,0%
	2	Count	5	2	7
		% within PR	9,3%	8,3%	9,0%
Total		Count	54	24	78
		% within PR	100,0%	100,0%	100,0%

a. Quart_age = 3

Chi-Square Tests[c]

	Value	df	Asymp. Sig. (2-sided)	Exact Sig. (2-sided)	Exact Sig. (1-sided)
Pearson Chi-Square	,017[a]	1	,895		
Continuity Correction[b]	,000	1	1,000		
Likelihood Ratio	,018	1	,894		
Fisher's Exact Test				1,000	,632
N of Valid Cases	78				

a. 2 cells (50,0%) have expected count less than 5. The minimum expected count is 2,15.

b. Computed only for a 2x2 table

c. Quart_age = 3

Risk Estimate[a]

	Value	95% Confidence Interval	
		Lower	Upper
Odds Ratio for RECNLR (1 / 2)	,891	,160	4,952
For cohort PR = 0	,966	,590	1,583
For cohort PR = 1	1,085	,320	3,680
N of Valid Cases	78		

a. Quart_age = 3

Quart_age = 4

Case Processing Summary[a]

	Cases					
	Valid		Missing		Total	
	N	Percent	N	Percent	N	Percent
RECNLR * PR	91	100,0%	0	,0%	91	100,0%

a. Quart_age = 4

RECNLR * PR Crosstabulation[a]

			PR		
			0	1	Total
RECNLR	1	Count	56	27	83
		% within PR	93,3%	87,1%	91,2%
	2	Count	4	4	8
		% within PR	6,7%	12,9%	8,8%
Total		Count	60	31	91
		% within PR	100,0%	100,0%	100,0%

a. Quart_age = 4

Chi-Square Tests[c]

	Value	df	Asymp. Sig. (2-sided)	Exact Sig. (2-sided)	Exact Sig. (1-sided)
Pearson Chi-Square	,991[a]	1	,319		
Continuity Correction[b]	,366	1	,545		
Likelihood Ratio	,945	1	,331		
Fisher's Exact Test				,437	,266
N of Valid Cases	91				

a. 1 cells (25,0%) have expected count less than 5. The minimum expected count is 2,73.

b. Computed only for a 2x2 table

Chi-Square Tests[c]

	Value	df	Asymp. Sig. (2-sided)	Exact Sig. (2-sided)	Exact Sig. (1-sided)
Pearson Chi-Square	,991[a]	1	,319		
Continuity Correction[b]	,366	1	,545		
Likelihood Ratio	,945	1	,331		
Fisher's Exact Test				,437	,266
N of Valid Cases	91				

a. 1 cells (25,0%) have expected count less than 5. The minimum expected count is 2,73.

b. Computed only for a 2x2 table

c. Quart_age = 4

Risk Estimate[a]

	Value	95% Confidence Interval	
		Lower	Upper
Odds Ratio for RECNLR (1 / 2)	2,074	,482	8,931
For cohort PR = 0	1,349	,664	2,742
For cohort PR = 1	,651	,305	1,390
N of Valid Cases	91		

a. Quart_age = 4

Quart_age = 5

Case Processing Summary[a]

	Cases					
	Valid		Missing		Total	
	N	Percent	N	Percent	N	Percent
RECNLR * PR	50	100,0%	0	,0%	50	100,0%

a. Quart_age = 5

RECNLR * PR Crosstabulation[a]

			PR		
			0	1	Total
RECNLR	1	Count	31	13	44
		% within PR	86,1%	92,9%	88,0%
	2	Count	5	1	6
		% within PR	13,9%	7,1%	12,0%
Total		Count	36	14	50
		% within PR	100,0%	100,0%	100,0%

a. Quart_age = 5

Chi-Square Tests[c]

	Value	df	Asymp. Sig. (2-sided)	Exact Sig. (2-sided)	Exact Sig. (1-sided)
Pearson Chi-Square	,434[a]	1	,510		
Continuity Correction[b]	,030	1	,861		
Likelihood Ratio	,476	1	,490		
Fisher's Exact Test				,663	,455
N of Valid Cases	50				

a. 2 cells (50,0%) have expected count less than 5. The minimum expected count is 1,68.

b. Computed only for a 2x2 table

c. Quart_age = 5

Risk Estimate[a]

	Value	95% Confidence Interval	
		Lower	Upper
Odds Ratio for RECNLR (1 / 2)	,477	,051	4,491
For cohort PR = 0	,845	,563	1,269
For cohort PR = 1	1,773	,280	11,234
N of Valid Cases	50		

a. Quart_age = 5

Quart_age = 6

Case Processing Summary[a]

	Cases					
	Valid		Missing		Total	
	N	Percent	N	Percent	N	Percent
RECNLR * PR	19	100,0%	0	,0%	19	100,0%

a. Quart_age = 6

RECNLR * PR Crosstabulation[a]

			PR		Total
			0	1	
RECNLR	1	Count	9	6	15
		% within PR	69,2%	100,0%	78,9%
	2	Count	4	0	4
		% within PR	30,8%	,0%	21,1%
Total		Count	13	6	19
		% within PR	100,0%	100,0%	100,0%

a. Quart_age = 6

Chi-Square Tests[c]

	Value	df	Asymp. Sig. (2-sided)	Exact Sig. (2-sided)	Exact Sig. (1-sided)
Pearson Chi-Square	2,338[a]	1	,126		
Continuity Correction[b]	,854	1	,356		
Likelihood Ratio	3,509	1	,061		
Fisher's Exact Test				,255	,184
N of Valid Cases	19				

a. 3 cells (75,0%) have expected count less than 5. The minimum expected count is 1,26.

b. Computed only for a 2x2 table

c. Quart_age = 6

Risk Estimate[a]

	Value	95% Confidence Interval	
		Lower	Upper
For cohort PR = 0	,600	,397	,907
N of Valid Cases	19		

a. Quart_age = 6

```
SORT CASES  BY menopausia.
SPLIT FILE SEPARATE BY menopausia.
CROSSTABS
  /TABLES=RECNLR BY PR
  /FORMAT=AVALUE TABLES
  /STATISTICS=CHISQ RISK
  /CELLS=COUNT COLUMN
  /COUNT ROUND CELL.
```

Crosstabs

Notes

Output Created		25-oct-2016 14:27:40
Comments		
Input	Data	C:\Users\evasquez\Documents\2016\Mastologia\NRI.sav
	Active Dataset	DataSet1
	Filter	<none>
	Weight	<none>
	Split File	menopausia
	N of Rows in Working Data File	288
Missing Value Handling	Definition of Missing	User-defined missing values are treated as missing.
	Cases Used	Statistics for each table are based on all the cases with valid data in the specified range(s) for all variables in each table.

Syntax		CROSSTABS<br>/TABLES=RECNLR BY PR<br>/FORMAT=AVALUE TABLES<br>/STATISTICS=CHISQ RISK<br>/CELLS=COUNT COLUMN<br>/COUNT ROUND CELL.
Resources	Processor Time	00:00:00,016
	Elapsed Time	00:00:00,016
	Dimensions Requested	2
	Cells Available	174762

[DataSet1] C:\Users\evasquez\Documents\2016\Mastologia\NRI.sav

menopausia = 0

Case Processing Summary[a]

	Cases					
	Valid		Missing		Total	
	N	Percent	N	Percent	N	Percent
RECNLR * PR	174	100,0%	0	,0%	174	100,0%

a. menopausia = 0

RECNLR * PR Crosstabulation[a]

			PR		Total
			0	1	
RECNLR	1	Count	111	47	158
		% within PR	92,5%	87,0%	90,8%
	2	Count	9	7	16
		% within PR	7,5%	13,0%	9,2%
Total		Count	120	54	174

	% within PR	100,0%	100,0%	100,0%

a. menopausia = 0

Chi-Square Tests[c]

	Value	df	Asymp. Sig. (2-sided)	Exact Sig. (2-sided)	Exact Sig. (1-sided)
Pearson Chi-Square	1,331[a]	1	,249		
Continuity Correction[b]	,757	1	,384		
Likelihood Ratio	1,262	1	,261		
Fisher's Exact Test				,265	,190
N of Valid Cases	174				

a. 1 cells (25,0%) have expected count less than 5. The minimum expected count is 4,97.

b. Computed only for a 2x2 table

c. menopausia = 0

Risk Estimate[a]

	Value	95% Confidence Interval	
		Lower	Upper
Odds Ratio for RECNLR (1 / 2)	1,837	,646	5,223
For cohort PR = 0	1,249	,801	1,947
For cohort PR = 1	,680	,371	1,245
N of Valid Cases	174		

a. menopausia = 0

menopausia = 1

Case Processing Summary[a]

	Cases					
	Valid		Missing		Total	
	N	Percent	N	Percent	N	Percent
RECNLR * PR	114	100,0%	0	,0%	114	100,0%

Case Processing Summary[a]

	Cases					
	Valid		Missing		Total	
	N	Percent	N	Percent	N	Percent
RECNLR * PR	114	100,0%	0	,0%	114	100,0%

a. menopausia = 1

RECNLR * PR Crosstabulation[a]

			PR		
			0	1	Total
RECNLR	1	Count	67	34	101
		% within PR	84,8%	97,1%	88,6%
	2	Count	12	1	13
		% within PR	15,2%	2,9%	11,4%
Total		Count	79	35	114
		% within PR	100,0%	100,0%	100,0%

a. menopausia = 1

Chi-Square Tests[c]

	Value	df	Asymp. Sig. (2-sided)	Exact Sig. (2-sided)	Exact Sig. (1-sided)
Pearson Chi-Square	3,651[a]	1	,056		
Continuity Correction[b]	2,533	1	,112		
Likelihood Ratio	4,522	1	,033		
Fisher's Exact Test				,063	,047
N of Valid Cases	114				

a. 1 cells (25,0%) have expected count less than 5. The minimum expected count is 3,99.

b. Computed only for a 2x2 table

c. menopausia = 1

Risk Estimate[a]

	Value	95% Confidence Interval	
		Lower	Upper

Odds Ratio for RECNLR (1 / 2)	,164	,020	1,316
For cohort PR = 0	,719	,583	,886
For cohort PR = 1	4,376	,653	29,343
N of Valid Cases	114		

a. menopausia = 1

```
SORT CASES  BY histo.
SPLIT FILE SEPARATE BY histo.
CROSSTABS
  /TABLES=RECNLR BY PR
  /FORMAT=AVALUE TABLES
  /STATISTICS=CHISQ RISK
  /CELLS=COUNT COLUMN
  /COUNT ROUND CELL.
```

Crosstabs

Notes

Output Created		25-oct-2016 14:28:36
Comments		
Input	Data	C:\Users\evasquez\Documents\2016\Mastologia\NRI.sav
	Active Dataset	DataSet1
	Filter	<none>
	Weight	<none>
	Split File	histo
	N of Rows in Working Data File	288
Missing Value Handling	Definition of Missing	User-defined missing values are treated as missing.
	Cases Used	Statistics for each table are based on all the cases with valid data in the specified range(s) for all variables in each table.

Syntax		CROSSTABS<br>/TABLES=RECNLR BY PR<br>/FORMAT=AVALUE TABLES<br>/STATISTICS=CHISQ RISK<br>/CELLS=COUNT COLUMN<br>/COUNT ROUND CELL.
Resources	Processor Time	00:00:00,032
	Elapsed Time	00:00:00,016
	Dimensions Requested	2
	Cells Available	174762

```
[DataSet1] C:\Users\evasquez\Documents\2016\Mastologia\NRI.sav
```

Warnings

No measures of association are computed for the crosstabulation of RECNLR * PR for split file histo= 2. At least one variable in each 2-way table upon which measures of association are computed is a constant.

histo = 1

Case Processing Summary[a]

	Cases					
	Valid		Missing		Total	
	N	Percent	N	Percent	N	Percent
RECNLR * PR	274	100,0%	0	,0%	274	100,0%

a. histo = 1

RECNLR * PR Crosstabulation[a]

	PR	Total

			0	1	
RECNLR	1	Count	170	76	246
		% within PR	89,0%	91,6%	89,8%
	2	Count	21	7	28
		% within PR	11,0%	8,4%	10,2%
Total		Count	191	83	274
		% within PR	100,0%	100,0%	100,0%

a. histo = 1

Chi-Square Tests[c]

	Value	df	Asymp. Sig. (2-sided)	Exact Sig. (2-sided)	Exact Sig. (1-sided)
Pearson Chi-Square	,414[a]	1	,520		
Continuity Correction[b]	,182	1	,670		
Likelihood Ratio	,427	1	,513		
Fisher's Exact Test				,665	,342
N of Valid Cases	274				

a. 0 cells (,0%) have expected count less than 5. The minimum expected count is 8,48.

b. Computed only for a 2x2 table

c. histo = 1

Risk Estimate[a]

	Value	95% Confidence Interval	
		Lower	Upper
Odds Ratio for RECNLR (1 / 2)	,746	,304	1,829
For cohort PR = 0	,921	,732	1,159
For cohort PR = 1	1,236	,633	2,411
N of Valid Cases	274		

a. histo = 1

histo = 2

Case Processing Summary[a]

	Cases					
	Valid		Missing		Total	
	N	Percent	N	Percent	N	Percent
RECNLR * PR	6	100,0%	0	,0%	6	100,0%

a. histo = 2

RECNLR * PR Crosstabulation[a]

			PR		Total
			0	1	
RECNLR	1	Count	5	1	6
		% within PR	100,0%	100,0%	100,0%
Total		Count	5	1	6
		% within PR	100,0%	100,0%	100,0%

a. histo = 2

Chi-Square Tests[b]

	Value
Pearson Chi-Square	.[a]
N of Valid Cases	6

a. No statistics are computed because RECNLR is a constant.

b. histo = 2

Risk Estimate[b]

	Value
Odds Ratio for RECNLR (1 / ÿÿÿÿÿÿïÿ)	.[a]

a. No statistics are computed because RECNLR is a constant.

b. histo = 2

histo = 3

Case Processing Summary[a]

	Cases					
	Valid		Missing		Total	
	N	Percent	N	Percent	N	Percent
RECNLR * PR	8	100,0%	0	,0%	8	100,0%

a. histo = 3

RECNLR * PR Crosstabulation[a]

			PR		Total
			0	1	
RECNLR	1	Count	3	4	7
		% within PR	100,0%	80,0%	87,5%
	2	Count	0	1	1
		% within PR	,0%	20,0%	12,5%
Total		Count	3	5	8
		% within PR	100,0%	100,0%	100,0%

a. histo = 3

Chi-Square Tests[c]

	Value	df	Asymp. Sig. (2-sided)	Exact Sig. (2-sided)	Exact Sig. (1-sided)
Pearson Chi-Square	,686[a]	1	,408		
Continuity Correction[b]	,000	1	1,000		
Likelihood Ratio	1,024	1	,312		
Fisher's Exact Test				1,000	,625
N of Valid Cases	8				

a. 4 cells (100,0%) have expected count less than 5. The minimum expected count is ,38.

b. Computed only for a 2x2 table

c. histo = 3

Risk Estimate[a]

	Value	95% Confidence Interval	
		Lower	Upper
For cohort PR = 1	,571	,301	1,085
N of Valid Cases	8		

a. histo = 3

```
SORT CASES  BY grado.
SPLIT FILE SEPARATE BY grado.
CROSSTABS
  /TABLES=RECNLR BY PR
  /FORMAT=AVALUE TABLES
  /STATISTICS=CHISQ RISK
  /CELLS=COUNT COLUMN
  /COUNT ROUND CELL.
```

Crosstabs

Notes

Output Created		25-oct-2016 14:29:00
Comments		
Input	Data	C:\Users\evasquez\Documents\2016\Mastologia\NRI.sav
	Active Dataset	DataSet1
	Filter	<none>
	Weight	<none>
	Split File	grado
	N of Rows in Working Data File	288
Missing Value Handling	Definition of Missing	User-defined missing values are treated as missing.

	Cases Used	Statistics for each table are based on all the cases with valid data in the specified range(s) for all variables in each table.
Syntax		CROSSTABS<br>/TABLES=RECNLR BY PR<br>/FORMAT=AVALUE TABLES<br>/STATISTICS=CHISQ RISK<br>/CELLS=COUNT COLUMN<br>/COUNT ROUND CELL.
Resources	Processor Time	00:00:00,015
	Elapsed Time	00:00:00,017
	Dimensions Requested	2
	Cells Available	174762

```
[DataSet1] C:\Users\evasquez\Documents\2016\Mastologia\NRI.sav
```

Warnings

No measures of association are computed for the crosstabulation of RECNLR * PR for split file grado= 1. At least one variable in each 2-way table upon which measures of association are computed is a constant.

grado = 0

Case Processing Summary[a]

	Cases					
	Valid		Missing		Total	
	N	Percent	N	Percent	N	Percent
RECNLR * PR	20	100,0%	0	,0%	20	100,0%

a. grado = 0

RECNLR * PR Crosstabulation[a]

			PR		Total
			0	1	
RECNLR	1	Count	12	7	19
		% within PR	100,0%	87,5%	95,0%
	2	Count	0	1	1
		% within PR	,0%	12,5%	5,0%
Total		Count	12	8	20
		% within PR	100,0%	100,0%	100,0%

a. grado = 0

Chi-Square Tests[c]

	Value	df	Asymp. Sig. (2-sided)	Exact Sig. (2-sided)	Exact Sig. (1-sided)
Pearson Chi-Square	1,579[a]	1	,209		
Continuity Correction[b]	,044	1	,834		
Likelihood Ratio	1,912	1	,167		
Fisher's Exact Test				,400	,400
N of Valid Cases	20				

a. 2 cells (50,0%) have expected count less than 5. The minimum expected count is ,40.

b. Computed only for a 2x2 table

c. grado = 0

Risk Estimate[a]

	Value	95% Confidence Interval	
		Lower	Upper
For cohort PR = 1	,368	,204	,664
N of Valid Cases	20		

a. grado = 0

grado = 1

Case Processing Summary[a]

	Cases					
	Valid		Missing		Total	
	N	Percent	N	Percent	N	Percent
RECNLR * PR	21	100,0%	0	,0%	21	100,0%

a. grado = 1

RECNLR * PR Crosstabulation[a]

			PR	Total
			0	
RECNLR	1	Count	20	20
		% within PR	95,2%	95,2%
	2	Count	1	1
		% within PR	4,8%	4,8%
Total		Count	21	21
		% within PR	100,0%	100,0%

a. grado = 1

Chi-Square Tests[b]

	Value
Pearson Chi-Square	.[a]
N of Valid Cases	21

a. No statistics are computed because PR is a constant.

b. grado = 1

Risk Estimate[b]

	Value
Odds Ratio for RECNLR (1 / 2)	.[a]

a. No statistics are computed because PR is a constant.

Risk Estimate[b]

	Value
Odds Ratio for RECNLR (1 / 2)	.[a]

a. No statistics are computed because PR is a constant.

b. grado = 1

grado = 2

Case Processing Summary[a]

	Cases					
	Valid		Missing		Total	
	N	Percent	N	Percent	N	Percent
RECNLR * PR	131	100,0%	0	,0%	131	100,0%

a. grado = 2

RECNLR * PR Crosstabulation[a]

			PR		Total
			0	1	
RECNLR	1	Count	84	34	118
		% within PR	91,3%	87,2%	90,1%
	2	Count	8	5	13
		% within PR	8,7%	12,8%	9,9%
Total		Count	92	39	131
		% within PR	100,0%	100,0%	100,0%

a. grado = 2

Chi-Square Tests[c]

	Value	df	Asymp. Sig. (2-sided)	Exact Sig. (2-sided)	Exact Sig. (1-sided)

Pearson Chi-Square	,521[a]	1	,470		
Continuity Correction[b]	,162	1	,687		
Likelihood Ratio	,500	1	,480		
Fisher's Exact Test				,527	,333
N of Valid Cases	131				

a. 1 cells (25,0%) have expected count less than 5. The minimum expected count is 3,87.

b. Computed only for a 2x2 table

c. grado = 2

Risk Estimate[a]

	Value	95% Confidence Interval	
		Lower	Upper
Odds Ratio for RECNLR (1 / 2)	1,544	,472	5,057
For cohort PR = 0	1,157	,741	1,805
For cohort PR = 1	,749	,356	1,576
N of Valid Cases	131		

a. grado = 2

grado = 3

Case Processing Summary[a]

	Cases					
	Valid		Missing		Total	
	N	Percent	N	Percent	N	Percent
RECNLR * PR	116	100,0%	0	,0%	116	100,0%

a. grado = 3

RECNLR * PR Crosstabulation[a]

			PR		Total
			0	1	
RECNLR	1	Count	62	40	102

		% within PR	83,8%	95,2%	87,9%
	2	Count	12	2	14
		% within PR	16,2%	4,8%	12,1%
Total		Count	74	42	116
		% within PR	100,0%	100,0%	100,0%

a. grado = 3

Chi-Square Tests[c]

	Value	df	Asymp. Sig. (2-sided)	Exact Sig. (2-sided)	Exact Sig. (1-sided)
Pearson Chi-Square	3,312[a]	1	,069		
Continuity Correction[b]	2,321	1	,128		
Likelihood Ratio	3,764	1	,052		
Fisher's Exact Test				,081	,059
N of Valid Cases	116				

a. 0 cells (,0%) have expected count less than 5. The minimum expected count is 5,07.

b. Computed only for a 2x2 table

c. grado = 3

Risk Estimate[a]

	Value	95% Confidence Interval	
		Lower	Upper
Odds Ratio for RECNLR (1 / 2)	,258	,055	1,216
For cohort PR = 0	,709	,544	,924
For cohort PR = 1	2,745	,744	10,130
N of Valid Cases	116		

a. grado = 3

```
SORT CASES  BY ILV.
SPLIT FILE SEPARATE BY ILV.
CROSSTABS
  /TABLES=RECNLR BY PR
  /FORMAT=AVALUE TABLES
  /STATISTICS=CHISQ RISK
  /CELLS=COUNT COLUMN
  /COUNT ROUND CELL.
```

Crosstabs

Notes

Output Created		25-oct-2016 14:29:22
Comments		
Input	Data	C:\Users\evasquez\Documents\2016\Mastologia\NRI.sav
	Active Dataset	DataSet1
	Filter	<none>
	Weight	<none>
	Split File	ILV
	N of Rows in Working Data File	288
Missing Value Handling	Definition of Missing	User-defined missing values are treated as missing.
	Cases Used	Statistics for each table are based on all the cases with valid data in the specified range(s) for all variables in each table.
Syntax		CROSSTABS<br>/TABLES=RECNLR BY PR<br>/FORMAT=AVALUE TABLES<br>/STATISTICS=CHISQ RISK<br>/CELLS=COUNT COLUMN<br>/COUNT ROUND CELL.
Resources	Processor Time	00:00:00,016
	Elapsed Time	00:00:00,016
	Dimensions Requested	2
	Cells Available	174762

```
[DataSet1] C:\Users\evasquez\Documents\2016\Mastologia\NRI.sav
```

ILV = 0

Case Processing Summary[a]

	Cases					
	Valid		Missing		Total	
	N	Percent	N	Percent	N	Percent
RECNLR * PR	98	100,0%	0	,0%	98	100,0%

a. ILV = 0

RECNLR * PR Crosstabulation[a]

			PR		
			0	1	Total
RECNLR	1	Count	73	16	89
		% within PR	91,3%	88,9%	90,8%
	2	Count	7	2	9
		% within PR	8,8%	11,1%	9,2%
Total		Count	80	18	98
		% within PR	100,0%	100,0%	100,0%

a. ILV = 0

Chi-Square Tests[c]

	Value	df	Asymp. Sig. (2-sided)	Exact Sig. (2-sided)	Exact Sig. (1-sided)
Pearson Chi-Square	,098[a]	1	,754		
Continuity Correction[b]	,000	1	1,000		
Likelihood Ratio	,094	1	,759		
Fisher's Exact Test				,668	,521
N of Valid Cases	98				

a. 1 cells (25,0%) have expected count less than 5. The minimum expected count is 1,65.

b. Computed only for a 2x2 table

Chi-Square Tests[c]

	Value	df	Asymp. Sig. (2-sided)	Exact Sig. (2-sided)	Exact Sig. (1-sided)
Pearson Chi-Square	,098[a]	1	,754		
Continuity Correction[b]	,000	1	1,000		
Likelihood Ratio	,094	1	,759		
Fisher's Exact Test				,668	,521
N of Valid Cases	98				

a. 1 cells (25,0%) have expected count less than 5. The minimum expected count is 1,65.

b. Computed only for a 2x2 table

c. ILV = 0

Risk Estimate[a]

	Value	95% Confidence Interval	
		Lower	Upper
Odds Ratio for RECNLR (1 / 2)	1,304	,247	6,870
For cohort PR = 0	1,055	,734	1,515
For cohort PR = 1	,809	,220	2,969
N of Valid Cases	98		

a. ILV = 0

ILV = 1

Case Processing Summary[a]

	Cases					
	Valid		Missing		Total	
	N	Percent	N	Percent	N	Percent
RECNLR * PR	90	100,0%	0	,0%	90	100,0%

a. ILV = 1

RECNLR * PR Crosstabulation[a]

			PR		Total
			0	1	
RECNLR	1	Count	65	17	82
		% within PR	90,3%	94,4%	91,1%
	2	Count	7	1	8
		% within PR	9,7%	5,6%	8,9%
Total		Count	72	18	90
		% within PR	100,0%	100,0%	100,0%

a. ILV = 1

Chi-Square Tests[c]

	Value	df	Asymp. Sig. (2-sided)	Exact Sig. (2-sided)	Exact Sig. (1-sided)
Pearson Chi-Square	,309[a]	1	,578		
Continuity Correction[b]	,009	1	,926		
Likelihood Ratio	,342	1	,559		
Fisher's Exact Test				1,000	,496
N of Valid Cases	90				

a. 1 cells (25,0%) have expected count less than 5. The minimum expected count is 1,60.

b. Computed only for a 2x2 table

c. ILV = 1

Risk Estimate[a]

	Value	95% Confidence Interval	
		Lower	Upper
Odds Ratio for RECNLR (1 / 2)	,546	,063	4,747
For cohort PR = 0	,906	,682	1,204
For cohort PR = 1	1,659	,253	10,887
N of Valid Cases	90		

a. ILV = 1

ILV = 2

Case Processing Summary[a]

	Cases					
	Valid		Missing		Total	
	N	Percent	N	Percent	N	Percent
RECNLR * PR	100	100,0%	0	,0%	100	100,0%

a. ILV = 2

RECNLR * PR Crosstabulation[a]

			PR		
			0	1	Total
RECNLR	1	Count	40	48	88
		% within PR	85,1%	90,6%	88,0%
	2	Count	7	5	12
		% within PR	14,9%	9,4%	12,0%
Total		Count	47	53	100
		% within PR	100,0%	100,0%	100,0%

a. ILV = 2

Chi-Square Tests[c]

	Value	df	Asymp. Sig. (2-sided)	Exact Sig. (2-sided)	Exact Sig. (1-sided)
Pearson Chi-Square	,703[a]	1	,402		
Continuity Correction[b]	,281	1	,596		
Likelihood Ratio	,703	1	,402		
Fisher's Exact Test				,540	,298
N of Valid Cases	100				

a. 0 cells (,0%) have expected count less than 5. The minimum expected count is 5,64.

b. Computed only for a 2x2 table

c. ILV = 2

Risk Estimate[a]

	Value	95% Confidence Interval	
		Lower	Upper
Odds Ratio for RECNLR (1 / 2)	,595	,175	2,020
For cohort PR = 0	,779	,459	1,324
For cohort PR = 1	1,309	,653	2,626
N of Valid Cases	100		

a. ILV = 2

```
SORT CASES  BY RE.
SPLIT FILE SEPARATE BY RE.
CROSSTABS
  /TABLES=RECNLR BY PR
  /FORMAT=AVALUE TABLES
  /STATISTICS=CHISQ RISK
  /CELLS=COUNT COLUMN
  /COUNT ROUND CELL.
```

Crosstabs

Notes

Output Created		25-oct-2016 14:29:46
Comments		
Input	Data	C:\Users\evasquez\Documents\2016\Mastologia\NRI.sav
	Active Dataset	DataSet1
	Filter	<none>
	Weight	<none>
	Split File	RE
	N of Rows in Working Data File	288
Missing Value Handling	Definition of Missing	User-defined missing values are treated as missing.

	Cases Used	Statistics for each table are based on all the cases with valid data in the specified range(s) for all variables in each table.
Syntax		CROSSTABS<br>/TABLES=RECNLR BY PR<br>/FORMAT=AVALUE TABLES<br>/STATISTICS=CHISQ RISK<br>/CELLS=COUNT COLUMN<br>/COUNT ROUND CELL.
Resources	Processor Time	00:00:00,016
	Elapsed Time	00:00:00,015
	Dimensions Requested	2
	Cells Available	174762

```
[DataSet1] C:\Users\evasquez\Documents\2016\Mastologia\NRI.sav
```

RE = 0

Case Processing Summary[a]

	Cases					
	Valid		Missing		Total	
	N	Percent	N	Percent	N	Percent
RECNLR * PR	110	100,0%	0	,0%	110	100,0%

a. RE = 0

RECNLR * PR Crosstabulation[a]

			PR		
			0	1	Total
RECNLR	1	Count	49	46	95
		% within PR	83,1%	90,2%	86,4%

	2	Count	10	5	15
		% within PR	16,9%	9,8%	13,6%
Total		Count	59	51	110
		% within PR	100,0%	100,0%	100,0%

a. RE = 0

Chi-Square Tests[c]

	Value	df	Asymp. Sig. (2-sided)	Exact Sig. (2-sided)	Exact Sig. (1-sided)
Pearson Chi-Square	1,186[a]	1	,276		
Continuity Correction[b]	,657	1	,418		
Likelihood Ratio	1,211	1	,271		
Fisher's Exact Test				,404	,210
N of Valid Cases	110				

a. 0 cells (,0%) have expected count less than 5. The minimum expected count is 6,95.

b. Computed only for a 2x2 table

c. RE = 0

Risk Estimate[a]

	Value	95% Confidence Interval	
		Lower	Upper
Odds Ratio for RECNLR (1 / 2)	,533	,169	1,676
For cohort PR = 0	,774	,515	1,163
For cohort PR = 1	1,453	,690	3,060
N of Valid Cases	110		

a. RE = 0

RE = 1

Case Processing Summary[a]

	Cases

	Valid		Missing		Total	
	N	Percent	N	Percent	N	Percent
RECNLR * PR	178	100,0%	0	,0%	178	100,0%

a. RE = 1

RECNLR * PR Crosstabulation[a]

			PR		Total
			0	1	
RECNLR	1	Count	129	35	164
		% within PR	92,1%	92,1%	92,1%
	2	Count	11	3	14
		% within PR	7,9%	7,9%	7,9%
Total		Count	140	38	178
		% within PR	100,0%	100,0%	100,0%

a. RE = 1

Chi-Square Tests[c]

	Value	df	Asymp. Sig. (2-sided)	Exact Sig. (2-sided)	Exact Sig. (1-sided)
Pearson Chi-Square	,000[a]	1	,994		
Continuity Correction[b]	,000	1	1,000		
Likelihood Ratio	,000	1	,994		
Fisher's Exact Test				1,000	,609
N of Valid Cases	178				

a. 1 cells (25,0%) have expected count less than 5. The minimum expected count is 2,99.

b. Computed only for a 2x2 table

c. RE = 1

Risk Estimate[a]

	Value	95% Confidence Interval	
		Lower	Upper
Odds Ratio for RECNLR (1 / 2)	1,005	,266	3,801

For cohort PR = 0	1,001	,753	1,331
For cohort PR = 1	,996	,350	2,832
N of Valid Cases	178		

a. RE = 1

```
SORT CASES  BY IMC2.
SPLIT FILE SEPARATE BY IMC2.
CROSSTABS
  /TABLES=RECNLR BY PR
  /FORMAT=AVALUE TABLES
  /STATISTICS=CHISQ RISK
  /CELLS=COUNT COLUMN
  /COUNT ROUND CELL.
```

Crosstabs

Notes

Output Created		25-oct-2016 14:31:09
Comments		
Input	Data	C:\Users\evasquez\Documents\2016\Mastologia\NRI.sav
	Active Dataset	DataSet1
	Filter	<none>
	Weight	<none>
	Split File	IMC2
	N of Rows in Working Data File	288
Missing Value Handling	Definition of Missing	User-defined missing values are treated as missing.
	Cases Used	Statistics for each table are based on all the cases with valid data in the specified range(s) for all variables in each table.

Syntax		CROSSTABS<br>/TABLES=RECNLR BY PR<br>/FORMAT=AVALUE TABLES<br>/STATISTICS=CHISQ RISK<br>/CELLS=COUNT COLUMN<br>/COUNT ROUND CELL.
Resources	Processor Time	00:00:00,000
	Elapsed Time	00:00:00,000
	Dimensions Requested	2
	Cells Available	174762

```
[DataSet1] C:\Users\evasquez\Documents\2016\Mastologia\NRI.sav
```

IMC2 = 1

Case Processing Summary[a]

	Cases					
	Valid		Missing		Total	
	N	Percent	N	Percent	N	Percent
RECNLR * PR	6	100,0%	0	,0%	6	100,0%

a. IMC2 = 1

RECNLR * PR Crosstabulation[a]

			PR		
			0	1	Total
RECNLR	1	Count	2	2	4
		% within PR	66,7%	66,7%	66,7%
	2	Count	1	1	2
		% within PR	33,3%	33,3%	33,3%
Total		Count	3	3	6

	% within PR	100,0%	100,0%	100,0%

a. IMC2 = 1

Chi-Square Tests[c]

	Value	df	Asymp. Sig. (2-sided)	Exact Sig. (2-sided)	Exact Sig. (1-sided)
Pearson Chi-Square	,000[a]	1	1,000		
Continuity Correction[b]	,000	1	1,000		
Likelihood Ratio	,000	1	1,000		
Fisher's Exact Test				1,000	,800
N of Valid Cases	6				

a. 4 cells (100,0%) have expected count less than 5. The minimum expected count is 1,00.

b. Computed only for a 2x2 table

c. IMC2 = 1

Risk Estimate[a]

	Value	95% Confidence Interval	
		Lower	Upper
Odds Ratio for RECNLR (1 / 2)	1,000	,034	29,807
For cohort PR = 0	1,000	,183	5,460
For cohort PR = 1	1,000	,183	5,460
N of Valid Cases	6		

a. IMC2 = 1

IMC2 = 2

Case Processing Summary[a]

	Cases					
	Valid		Missing		Total	
	N	Percent	N	Percent	N	Percent
RECNLR * PR	87	100,0%	0	,0%	87	100,0%

Case Processing Summary[a]

	Cases					
	Valid		Missing		Total	
	N	Percent	N	Percent	N	Percent
RECNLR * PR	87	100,0%	0	,0%	87	100,0%

a. IMC2 = 2

RECNLR * PR Crosstabulation[a]

			PR		Total
			0	1	
RECNLR	1	Count	61	21	82
		% within PR	93,8%	95,5%	94,3%
	2	Count	4	1	5
		% within PR	6,2%	4,5%	5,7%
Total		Count	65	22	87
		% within PR	100,0%	100,0%	100,0%

a. IMC2 = 2

Chi-Square Tests[c]

	Value	df	Asymp. Sig. (2-sided)	Exact Sig. (2-sided)	Exact Sig. (1-sided)
Pearson Chi-Square	,078[a]	1	,779		
Continuity Correction[b]	,000	1	1,000		
Likelihood Ratio	,082	1	,774		
Fisher's Exact Test				1,000	,627
N of Valid Cases	87				

a. 2 cells (50,0%) have expected count less than 5. The minimum expected count is 1,26.

b. Computed only for a 2x2 table

c. IMC2 = 2

Risk Estimate[a]

	Value	95% Confidence Interval	
		Lower	Upper

Odds Ratio for RECNLR (1 / 2)	,726	,077	6,867
For cohort PR = 0	,930	,589	1,468
For cohort PR = 1	1,280	,213	7,680
N of Valid Cases	87		

a. IMC2 = 2

IMC2 = 3

Case Processing Summary[a]

	Cases					
	Valid		Missing		Total	
	N	Percent	N	Percent	N	Percent
RECNLR * PR	122	100,0%	0	,0%	122	100,0%

a. IMC2 = 3

RECNLR * PR Crosstabulation[a]

			PR		
			0	1	Total
RECNLR	1	Count	69	41	110
		% within PR	88,5%	93,2%	90,2%
	2	Count	9	3	12
		% within PR	11,5%	6,8%	9,8%
Total		Count	78	44	122
		% within PR	100,0%	100,0%	100,0%

a. IMC2 = 3

Chi-Square Tests[c]

	Value	df	Asymp. Sig. (2-sided)	Exact Sig. (2-sided)	Exact Sig. (1-sided)
Pearson Chi-Square	,707[a]	1	,401		

Continuity Correction[b]	,275	1	,600		
Likelihood Ratio	,744	1	,388		
Fisher's Exact Test				,534	,307
N of Valid Cases	122				

a. 1 cells (25,0%) have expected count less than 5. The minimum expected count is 4,33.

b. Computed only for a 2x2 table

c. IMC2 = 3

Risk Estimate[a]

	Value	95% Confidence Interval	
		Lower	Upper
Odds Ratio for RECNLR (1 / 2)	,561	,144	2,191
For cohort PR = 0	,836	,585	1,195
For cohort PR = 1	1,491	,543	4,091
N of Valid Cases	122		

a. IMC2 = 3

IMC2 = 4

Case Processing Summary[a]

	Cases					
	Valid		Missing		Total	
	N	Percent	N	Percent	N	Percent
RECNLR * PR	73	100,0%	0	,0%	73	100,0%

a. IMC2 = 4

RECNLR * PR Crosstabulation[a]

			PR		Total
			0	1	
RECNLR	1	Count	46	17	63
		% within PR	86,8%	85,0%	86,3%

2	Count	7	3	10
	% within PR	13,2%	15,0%	13,7%
Total	Count	53	20	73
	% within PR	100,0%	100,0%	100,0%

a. IMC2 = 4

Chi-Square Tests[c]

	Value	df	Asymp. Sig. (2-sided)	Exact Sig. (2-sided)	Exact Sig. (1-sided)
Pearson Chi-Square	,039[a]	1	,843		
Continuity Correction[b]	,000	1	1,000		
Likelihood Ratio	,039	1	,844		
Fisher's Exact Test				1,000	,555
N of Valid Cases	73				

a. 1 cells (25,0%) have expected count less than 5. The minimum expected count is 2,74.

b. Computed only for a 2x2 table

c. IMC2 = 4

Risk Estimate[a]

	Value	95% Confidence Interval	
		Lower	Upper
Odds Ratio for RECNLR (1 / 2)	1,160	,269	5,006
For cohort PR = 0	1,043	,677	1,608
For cohort PR = 1	,899	,321	2,520
N of Valid Cases	73		

a. IMC2 = 4

```
SPLIT FILE OFF.
USE ALL.
COMPUTE filter_$=(menopausia = 1).
VARIABLE LABEL filter_$ 'menopausia = 1 (FILTER)'.
VALUE LABELS filter_$ 0 'Not Selected' 1 'Selected'.
FORMAT filter_$ (f1.0).
FILTER BY filter_$.
EXECUTE.
CROSSTABS
  /TABLES=RECNLR BY PR
```

```
/FORMAT=AVALUE TABLES
/STATISTICS=CHISQ RISK
/CELLS=COUNT COLUMN
/COUNT ROUND CELL.
```

Crosstabs

Notes

Output Created		25-oct-2016 14:32:09
Comments		
Input	Data	C:\Users\evasquez\Documents\2016\Mastologia\NRI.sav
	Active Dataset	DataSet1
	Filter	menopausia = 1 (FILTER)
	Weight	<none>
	Split File	<none>
	N of Rows in Working Data File	114
Missing Value Handling	Definition of Missing	User-defined missing values are treated as missing.
	Cases Used	Statistics for each table are based on all the cases with valid data in the specified range(s) for all variables in each table.
Syntax		CROSSTABS<br>/TABLES=RECNLR BY PR<br>/FORMAT=AVALUE TABLES<br>/STATISTICS=CHISQ RISK<br>/CELLS=COUNT COLUMN<br>/COUNT ROUND CELL.
Resources	Processor Time	00:00:00,016
	Elapsed Time	00:00:00,016
	Dimensions Requested	2
	Cells Available	174762

```
[DataSet1] C:\Users\evasquez\Documents\2016\Mastologia\NRI.sav
```

Case Processing Summary

	Cases					
	Valid		Missing		Total	
	N	Percent	N	Percent	N	Percent
RECNLR * PR	114	100,0%	0	,0%	114	100,0%

RECNLR * PR Crosstabulation

			PR		Total
			0	1	
RECNLR	1	Count	67	34	101
		% within PR	84,8%	97,1%	88,6%
	2	Count	12	1	13
		% within PR	15,2%	2,9%	11,4%
Total		Count	79	35	114
		% within PR	100,0%	100,0%	100,0%

Chi-Square Tests

	Value	df	Asymp. Sig. (2-sided)	Exact Sig. (2-sided)	Exact Sig. (1-sided)
Pearson Chi-Square	3,651[a]	1	,056		
Continuity Correction[b]	2,533	1	,112		
Likelihood Ratio	4,522	1	,033		
Fisher's Exact Test				,063	,047
N of Valid Cases	114				

a. 1 cells (25,0%) have expected count less than 5. The minimum expected count is 3,99.

b. Computed only for a 2x2 table

Risk Estimate

	Value	95% Confidence Interval

		Lower	Upper
Odds Ratio for RECNLR (1 / 2)	,164	,020	1,316
For cohort PR = 0	,719	,583	,886
For cohort PR = 1	4,376	,653	29,343
N of Valid Cases	114		

```
LOGISTIC REGRESSION VARIABLES PR
  /METHOD=FSTEP(COND) RECNLR histo grado ILV ajcc T_stage N RE RP
  /CONTRAST (RECNLR)=Indicator(1)
  /CONTRAST (histo)=Indicator(1)
  /CONTRAST (grado)=Indicator(1)
  /CONTRAST (ILV)=Indicator(1)
  /CONTRAST (ajcc)=Indicator(1)
  /PRINT=CI(95)
  /CRITERIA=PIN(0.05) POUT(0.10) ITERATE(20) CUT(0.5).
```

Logistic Regression

Notes

Output Created		25-oct-2016 14:35:20
Comments		
Input	Data	C:\Users\evasquez\Documents\2016\Mastologia\NRI.sav
	Active Dataset	DataSet1
	Filter	menopausia = 1 (FILTER)
	Weight	<none>
	Split File	<none>
	N of Rows in Working Data File	114
Missing Value Handling	Definition of Missing	User-defined missing values are treated as missing

Syntax		LOGISTIC REGRESSION VARIABLES PR<br>/METHOD=FSTEP(COND) RECNLR histo grado ILV ajcc T_stage N RE RP<br>/CONTRAST (RECNLR)=Indicator(1)<br>/CONTRAST (histo)=Indicator(1)<br>/CONTRAST (grado)=Indicator(1)<br>/CONTRAST (ILV)=Indicator(1)<br>/CONTRAST (ajcc)=Indicator(1)<br>/PRINT=CI(95)<br>/CRITERIA=PIN(0.05) POUT(0.10) ITERATE(20) CUT(0.5).
Resources	Processor Time	00:00:00,016
	Elapsed Time	00:00:00,016

```
[DataSet1] C:\Users\evasquez\Documents\2016\Mastologia\NRI.sav
```

Case Processing Summary

Unweighted Cases[a]		N	Percent
Selected Cases	Included in Analysis	114	100,0
	Missing Cases	0	,0
	Total	114	100,0
Unselected Cases		0	,0
Total		114	100,0

a. If weight is in effect, see classification table for the total number of cases.

b. The category variable histo is constant for the selected cases. Since a constant term was specified, the variable will be removed from the analysis.

c. The category variable grado is constant for the selected cases. Since a constant term was specified, the variable will be removed from the analysis.

d. The category variable ILV is constant for the selected cases. Since a constant term was specified, the variable will be removed from the analysis.

Case Processing Summary

Unweighted Cases[a]		N	Percent
Selected Cases	Included in Analysis	114	100,0
	Missing Cases	0	,0
	Total	114	100,0
Unselected Cases		0	,0
Total		114	100,0

a. If weight is in effect, see classification table for the total number of cases.

b. The category variable histo is constant for the selected cases. Since a constant term was specified, the variable will be removed from the analysis.

c. The category variable grado is constant for the selected cases. Since a constant term was specified, the variable will be removed from the analysis.

d. The category variable ILV is constant for the selected cases. Since a constant term was specified, the variable will be removed from the analysis.

e. The category variable ajcc is constant for the selected cases. Since a constant term was specified, the variable will be removed from the analysis.

Dependent Variable Encoding

Original Value	Internal Value
0	0
1	1

Categorical Variables Codings

		Frequency	Parameter coding (1)
RECNLR	1	101	,000
	2	13	1,000

Block 0: Beginning Block

Classification Table[a,b]

Observed			Predicted		
			PR		Percentage Correct
			0	1	
Step 0	PR	0	79	0	100,0
		1	35	0	,0
	Overall Percentage				69,3

a. Constant is included in the model.

b. The cut value is ,500

Variables in the Equation

		B	S.E.	Wald	df	Sig.	Exp(B)
Step 0	Constant	-,814	,203	16,075	1	,000	,443

Variables not in the Equation

			Score	df	Sig.
Step 0	Variables	RECNLR(1)	3,651	1	,056
		T_stage	2,935	1	,087
		N	2,204	1	,138
		RE	8,102	1	,004
		RP	5,017	1	,025
	Overall Statistics		15,288	5	,009

Block 1: Method = Forward Stepwise (Conditional)

Omnibus Tests of Model Coefficients

	Chi-square	df	Sig.

Step 1	Step	8,036	1	,005
	Block	8,036	1	,005
	Model	8,036	1	,005
Step 2	Step	5,337	1	,021
	Block	13,373	2	,001
	Model	13,373	2	,001

Model Summary

Step	-2 Log likelihood	Cox & Snell R Square	Nagelkerke R Square
1	132,570[a]	,068	,096
2	127,233[b]	,111	,156

a. Estimation terminated at iteration number 4 because parameter estimates changed by less than ,001.

b. Estimation terminated at iteration number 5 because parameter estimates changed by less than ,001.

Classification Table[a]

Observed			Predicted		
			PR		Percentage Correct
			0	1	
Step 1	PR	0	79	0	100,0
		1	35	0	,0
	Overall Percentage				69,3
Step 2	PR	0	60	19	75,9
		1	14	21	60,0
	Overall Percentage				71,1

a. The cut value is ,500

Variables in the Equation

		B	S.E.	Wald	df	Sig.	Exp(B)
Step 1[a]	RE	-1,176	,421	7,783	1	,005	,309
	Constant	-,174	,296	,347	1	,556	,840

Step 2[b]	RECNLR(1)	-1,997	1,081	3,409	1	,065	,136
	RE	-1,262	,433	8,508	1	,004	,283
	Constant	,027	,313	,007	1	,932	1,027

a. Variable(s) entered on step 1: RE.

b. Variable(s) entered on step 2: RECNLR.

Variables in the Equation

		95% C.I.for EXP(B)	
		Lower	Upper
Step 1[a]	RE	,135	,705
	Constant		
Step 2[b]	RECNLR(1)	,016	1,131
	RE	,121	,661
	Constant		

a. Variable(s) entered on step 1: RE.

b. Variable(s) entered on step 2: RECNLR.

Model if Term Removed[a]

Variable		Model Log Likelihood	Change in -2 Log Likelihood	df	Sig. of the Change
Step 1	RE	-70,347	8,123	1	,004
Step 2	RECNLR	-66,347	5,462	1	,019
	RE	-68,087	8,941	1	,003

a. Based on conditional parameter estimates

Variables not in the Equation

			Score	df	Sig.
Step 1	Variables	RECNLR(1)	4,358	1	,037
		T_stage	3,336	1	,068
		N	1,632	1	,201
		RP	,193	1	,661
	Overall Statistics		7,610	4	,107
Step 2	Variables	T_stage	2,277	1	,131
		N	1,380	1	,240

RP	,179	1	,672
Overall Statistics	3,456	3	,326
